# Fallout from the COVID-19 pandemic – should we prepare for a tsunami of post viral depression?

**DOI:** 10.1017/ipm.2020.40

**Published:** 2020-05-15

**Authors:** D. Lyons, M. Frampton, S. Naqvi, D. Donohoe, G. Adams, K. Glynn

**Affiliations:** 1 Department of Psychiatry of Later Life, St. Patrick’s Mental Health Services James’s St, Dublin 8, Ireland; 2 Department of Old Age Psychiatry, Carew House, St. Vincent’s University Hospital, Elm Park, Dublin 4, Ireland; 3 Department of Psychiatry, University Hospital Limerick, Limerick, Ireland

**Keywords:** Coronavirus, COVID-19, major depressive disorder, myalgic encephalomyelitis

## Abstract

The current COVID-19 pandemic is not just a medical and social tragedy, but within the threat of the outbreak looms the potential for a significant and persistent negative mental health impact, based on previous experience with other pandemics such as Severe Acute Respiratory Syndrome (SARS) in 2003 and the earlier H1N1 outbreak of 1918. This piece will highlight the links between depression and viral illnesses and explore important overlaps with myalgic encephalomyelitis/chronic fatigue syndrome, potentially implicating inflammatory mechanisms in those exposed to a range of viral agents. While containment of psychological distress currently focuses on social anxiety and quarantine measures, a second wave of psychological morbidity due to viral illness may be imminent.

## Introduction

For most of human, history infectious diseases were responsible for the greatest burden of premature death and morbidity, and global pandemics over the centuries have threatened the survival of entire populations. Notable outbreaks that were seared into collective memory owing to their associated mass casualties included diseases such as smallpox, cholera and influenza. Widespread immunization through safe and effective vaccine usage and increased deployment of antibiotics considerably reduced the toll of infectious diseases, at least in developed countries, by the middle of the 20th century. Emerging pandemic viral infections remain a constant threat to human health, however, many entering the human population (as is allegedly the case with COVID-19) from contact with animals (Holmes *et al*. 2017).

Compared with antibiotics to treat bacterial infection, relatively few antiviral drugs have been developed to treat emerging viral infections and their complications; therefore, breaking the chain of transmission is a crucial intervention in containing any outbreak of novel viruses. The unprecedented public health measures undertaken across the world, since China first reported cases of the novel Coronavirus in December 2019, have necessarily entailed significant social disruption and jeopardized the economic prospects of entire communities. While the negative psychological effects of prolonged quarantine measures may also seem obvious, does the recent outbreak of COVID-19 sweeping around the world potentially carry a second layer of psychological morbidity in the form of depression and mood disorder in its’ wake? This piece will consider how post viral psychogenic sequelae are conceptualized and highlight certain factors for clinical contemplation, once the acute infective phase of coronavirus has passed.

## The role of inflammation

Clinical or major depressive disorder interacts with disability and medical illness in a variety of ways that are complex and often with a bidirectional relationship, especially in respect of cardiovascular illness (Blazer & Hybels, 2005). The development of mood disorder has also been linked to inflammation (Howren *et al*. 2009), and experimental activation of inflammatory reactions has been demonstrated to induce symptoms of mood disorders in both human and animal studies (Eisenberger *et al*. 2010). In particular, decreased cellular immunity results in the formation of neuromodulators and cytokine peptides or interleukins, which are hypothesized to penetrate the brain when the blood– central nervous system (CNS) barrier is compromised during time of stress, infection and inflammation (Irani & Lang, 2008). Immune components such as proinflammatory cytokines and brain-reactive antibodies are theorized to induce changes in neurotransmitter and neuroendocrine function, such as hypercortisolism, and it has long been appreciated that cortisol hypersecretion is potentially related to a range of psychiatric disorders (Pivonello *et al*. 2015). Although the mechanisms for interaction between mental health difficulties and communicable diseases, namely infections, may still be the subject of speculation, in relation to specific triggers for psychiatric episodes, it seems not unreasonable to assume that they are far from being solely psychosocial in origin.

## Remembering ME

Myalgic encephalomyelitis (abbreviated to ME), but also known as chronic fatigue syndrome (CFS) is a complex, disabling chronic illness characterized by extreme fatigue that is not explained by any underlying medical condition, which is said to affect 0.76–3.28% of the world-wide population (Johnston *et al*. 2013). Symptoms constellations associated with ME include musculoskeletal pain, headaches, sore throat, tender lymph nodes, concentration and memory difficulties, unrefreshing sleep and exacerbation of these symptoms with what is felt to be the cardinal feature of the condition, namely post exertional malaise in response to minimal physical or cognitive exertion (Fukuda *et al*. 1994). The term ‘benign myalgic encephalomyelitis’ was first deployed in relation to what appeared to be an infective but low-mortality outbreak (in sporadic and epidemic fashion) at the Royal Free Hospital in London in the 1950s (Wojcik *et al*. 2011). By the 1980s, following a further outbreak of an illness resembling infectious mononucleosis in the United States, an initial link to the Epstein Barr virus was suggested and working groups established to reach consensus about diagnostic criteria, with Fukuda et al. finally publishing diagnostic criteria in 1994. The illness has remained somewhat controversial over the intervening years, with patient groups feeling the condition has been somewhat trivialized by medics, who failed to agree on the etiology, seriousness or prevalence of ME/CFS and patients themselves being frustrated by persistent professional reference to psychological components of CFS, which they rejected as offensive (Dumit, 2006). This was not helped by the popular media which initially, despite being supportive of efforts to raise awareness of ME/CFS and to highlight an organic attribution, subsequently nicknamed the condition the ‘yuppie flu’ in the 1990s.

## At risk of ME/chronic fatigue?

A recent review of peri-onset events reported by subjects meeting ME/CFS criteria identified the most common peri-onset events as being infection-related episodes (64%) as opposed to stressful incidents (34%) or exposure to environmental toxins (20%) (Chu *et al*. 2019). In their prospective, population-based cohort study in Denmark, Benros *et al*. (2013), using 78 million person years of follow-up drawn from Danish longitudinal registers, found that any history of hospitalization for infection increased the risk of mood disorders by 62% with many displaying the symptom of prominent fatigue as a hallmark. With 32% of their study participants who had mood disorder having had a previous hospital contact for an infection, they speculated that these associations seemed compatible with an immunologic hypothesis for the development of depression and mood disorder in subgroups of patients. The Danish group pointed to the symptom overlap emanating from systemic infection and depression and the symptoms common to both which they termed ‘sickness behavior’ including fatigue, apathy, reduced social interaction, impaired concentration and sleep disturbance, which they felt could become rather chronic and progress to major depression in some cases. Benros also noted that the number of infections and autoimmune disorders increased the risk of mood disorders in a dose–response relationship. ME/CFS is consistently more prevalent in females (who also have higher rates of autoimmune disorders) than in males, as 60–85% of all cases in the United States were women, most commonly aged between 40 and 60 years (Dinos *et al*. 2009). Psychological factors such as pre-existing depressive and anxiety disorders, perfectionistic personality type and a childhood trauma history were predisposing factors identified in a review by Lievesley *et al*. (2014).

## Links with which viruses?

Because ME/CFS may begin as a flu-like illness with a sudden onset, various infectious causes have been proposed right from the outset of clinical observation of this condition, but it should be emphasized that the exact pathogenic mechanism is unclear. A 2016 report by the Institute of Medicine (which is a US-based NGO) concludes that ME/CFS is a biologically based illness but that markers and abnormalities are not yet sensitive enough to be useful as a diagnosis (Unger *et al*. 2016). While in the majority of cases, there appears to be no conclusive evidence for chronic viral infection, it has been plausibly proposed that viruses could act via ‘a hit and run’ mechanism: this theory proposes that viruses trigger the disease, cause immune abnormalities and leave in their wake a dysfunctional immune system and/or autoimmunity (Rasa *et al*. 2018). Although various viral, and even microbial infections, are considered to be possible triggers for a subsequent diagnosis of ME/CFS, studies have been conducted on the association of ME/CFS with Epstein–Barr virus (EBV), cytomegalovirus (CMV), human herpesviruses type 6 and 7, human parvovirus and enteroviruses (Strauss *et al*. 1985; Holmes *et al*. 1987; Martin, 1997).

The Toronto-based psychiatrist and sleep specialist Henry Moldofsky studied the long-term adverse effects of the Severe Acute Respiratory Syndrome (SARS) on a subgroup of patients, the majority of whom were healthcare workers and who remained unable to return to their former occupation (Moldofsky & Patcai, 2011). SARS is a viral respiratory disease that surfaced in Asia in the early 2000s caused by the first identified strain of the SARS coronavirus species. Although the majority (93.5%) of the sickest patients admitted to hospitals in Toronto survived (Booth *et al*. 2003), longer term outcomes surveillance by Moldofsky’s group found a profile of symptoms such as daytime fatigue, myalgia, weakness and depression very reminiscent of ME/CFS in the cases that remained occupationally and functionally impaired. Of note, Moldofsky’s small sample of 22 cases (which represented only 8% of those who recovered from SARS) had not been exposed to lengthy periods of quarantine and had similar outcomes to a more widely selected, ostensibly recovered, population of Toronto SARS patients who were subsequently surveyed. In a larger study of 107 such patients, similar problems with pain, reduced vitality and impaired physical, mental and social functioning were revealed in up to 82% of patients, who had returned to unmodified work, 1-year post initial infection (Herridge *et al*. 2003).

The relevant literature (Wang *et al*. 2015) also purports to conclude that other viral agents are linked to depression and anxiety in developed countries, not only EBV, Borna virus disease and Varicella-Zoster virus but also human immunodeficiency virus (Van den Heuvel, 2013), influenza A (H1N1) (Manjunatha *et al*. 2011) and other influenza viruses. Coughlin (2012) in his review however acknowledges that frameworks for understanding linkages between mood disorder and anxiety are not yet sufficiently robust, but their further exploration offers potential for prevention of psychological distress through vaccination and via improved treatment of the viral illness directly, as is the case with Hepatitis C and HIV/AIDS. Gale et al. found the strongest associations between virus exposure and depression in a sample of US adults existing for subjects who were seropositive for Herpes Simplex Virus type-2, but to a lesser extent for CMV (Gale, 2018). They found no association with depression and Hepatitis A and B or herpes simplex type 1 infection.

## Coronavirus and depression and/or sickness behavior – a classic false dichotomy?

At the level of the pathogen–immune system interface, it is important to appreciate that there may be differences as well as similarities between sickness behavior and clinical depression (Dantzer, 2001). Symptoms such as fatigue, sleep and appetite disturbance, decreased social interaction and loss of interest in usual activities are seen in both clinical depression and sickness behavior related to viral infections (Vollmer-Conna, 2001). Clinically, however, the core psychological symptoms of depression (hopelessness, worthlessness, pessimism and guilt) would be more typical of depression than sickness alone (Gelder *et al*. 2001). Okusaga et al., while speculating on an infection to mood rather than mood to infection causality direction, highlighted an association between seropositivity for influenza and coronaviruses and a subsequent history of mood disorders. In addition, seropositivity for influenza B was concerningly associated with suicidal behavior and a lifetime history of psychotic symptoms in patients with mood disorders (Okusaga *et al*. 2011). It remains unclear whether the viruses themselves or the immune response to them are the main culprit in leading to mood disorder, but it is worth noting that both influenza and coronaviruses are potentially neurotropic and have been isolated from the CNS (Xu *et al*. 2005). Cytokines involved in the immune response against influenza infection enhance activation of the HPA axis as well as reportedly causing a depletion of tryptophan in the brain. In people who developed mood disorder post infection, Okusaga et al. failed to note neurological complications of viral illness or evidence of encephalitis due to direct effects of viral infection, reinforcing the view of an immune basis as being the main culprit leading to mood disorder in their sample.

When one considers the entire symptomatic spectrum associated with mood disorder – both emotional/cognitive and the full range of physical symptoms (which encompass the so-called sickness behavior which we more readily associate perhaps with infection), it becomes possible to re-conceptualize the diversity of mood disorder in terms of etiology and perhaps ultimately remediation. A Western conceptualization puts affective symptoms front and center, whereas non-Western patients who meet Diagnostic and Statistical Manual criteria for major depression report primarily somatic symptoms, reflecting in part cultural differences in the stigmatization of mental illness (Canli, 2014).

## Can 1918 teach us anything?

Comparisons are being currently made between the present COVID-19 pandemic and the so-called Spanish Flu pandemic of 1918–1919, as we try to acclimatize ourselves to the rapidly changing social circumstances of 2020. While little formal research was conducted on the long-term impact of the Spanish flu on mental health, Sven-Erik Mamelund (2010) studied asylum hospitalization during the period in question and found that the number of first-time hospitalizations due to influenza related mental disorders increased by an average annual factor of 7.2 in the 6 years after the pandemic. Spanish flu survivors reported sleep disturbances, depression, dizziness and difficulty coping at work, and increased death rates due to suicide were noted in the United States, according to Mamelund. Psychiatrists and neurologists first reported encountering encephalitis lethargica symptoms in 1916 in Austria and France but by 1919, it had become common throughout much of the World. Although many clinicians (at the time and subsequently) surmised an association between encephalitis lethargica and the Spanish flu, no conclusive evidence of causality exists (Beiner, 2006). Although the psychological reaction to the Spanish flu may have been either squeezed in terms of perceived importance by the conflict of World War I and its conclusion, if history teaches us anything it is to expect a swathe of mental health challenges following in tow of the present pandemic.

## Preparing for the worst – hoping for the best

As we have seen, the documented connection between viral pandemics and psychological stress is not new. It was the American psychiatrist Karl A. Menninger who urged colleagues to awaken from complacency in relation to the emerging connection between the 1918 pandemic and psychiatric complications, by realizing that although the influenza virus now as then most commonly affects the respiratory system, the burden on neuropsychiatric disease was under-recognized (Menninger, 1919). Yet the citizenry, of the developed and developing world alike, navigating the present pandemic are experiencing unique and profound economic shocks after a relative period of global prosperity, stability and peace. Developed economies are promising fiscal safety nets and stimulus to counter economic aftershocks, but few are reassured that they will be either sustainable or adequate in the medium to long term. For struggling health systems (and that appears to be the majority), it is immediate counter-measures to flatten the infective curve that take priority, and few if any, are giving consideration to or anticipating neuropsychiatric manifestations, that may take months or years to appear. Part of those counter-measures include mass quarantine to safeguard more vulnerable members of society and the negative psychological effects of self-isolation have received publicity and continue to attract clinical concern from public health officials who must balance the distress associated with restriction of liberty with acceleration of the spread of COVID-19. In a useful review by Brooks *et al*. (2020) with reference to the present pandemic, she and her colleagues argue for an appeal to altruism in respect of public compliance with restrictive measures, but that in turn, authorities should provide adequate information about the rationale for the lockdown and should only extend quarantine measures if absolutely necessary.

## What can psychiatrists do?

In the current phase of the pandemic, psychiatry could add a collective voice to quell the calls for premature easing of social distance and other mitigation measures, by reminding sceptics that not only may a reduction in mortality be achieved but also potentially many cases of neuropsychiatric illness, associated with significant long-term economic burden and individual distress. We can listen for and be attuned to the symptoms of post viral depression, when cases begin to appear and educate our colleagues about these conditions as genuine entities deserving of care, support and rehabilitation. In this way, perhaps many of the previous mistakes around interacting with individuals with ME/CFS can be avoided. In future, perhaps we will attempt to prevent neurotropic respiratory viral infections more aggressively by reducing risk factors such as smoking, obesity and the metabolic syndrome associated with many of our treatments. Those recovering from viral infections may need closer monitoring in terms of suicide risk and we may consider even prophylactic use of antidepressants for a brief time in treating patients at risk.

In the meantime, we must never cease to counsel those who have had to come to terms with socially unsupported grief or to reassure those who once regarded a nursing home as a safe sanctuary and who now experience a level of personal as well as collective insecurity, in fearing an undignified, lonely demise. We will do well to help people attempt to reframe the cocooning experience as a period of nurturing self-sufficiency and self-awareness, to talk up the benefits of routine and exercise, getting the correct balance of rest and activity for those recovering from viral infection to avoid exacerbating their fatigue. We should speak out on behalf of those with mental illness for access to basic subvention and supplies including medication. We will not only observe the present unplanned and unwelcome social experiences and share in many of them because of COVID-19 but also be mindful of factors such as mutual solidarity and resilience, adaptability, flexibility to work and interact differently through technology use – all of which will almost certainly lessen the impact of the current pandemic.

## Financial Support

This article received no specific grant from any funding agency, commercial or not-for-profit sectors.

## Conflict of interest

The authors have no conflict of interest to disclose

## Ethical Standards

The authors assert that all procedures contributing to this work comply with the ethical standards of the relevant national and institutional committee on human experimentation with the Helsinki Declaration of 1975, as revised in 2008. The authors assert that ethical approval was not required for publication of this manuscript.
